# A surfactant polymer wound dressing protects human keratinocytes from inducible necroptosis

**DOI:** 10.1038/s41598-021-82260-x

**Published:** 2021-02-23

**Authors:** Puneet Khandelwal, Amitava Das, Chandan K. Sen, Sangly P. Srinivas, Sashwati Roy, Savita Khanna

**Affiliations:** 1 Department of Surgery, Indiana Center for Regenerative Medicine and Engineering, Indiana University School of Medicine, 975 W Walnut St, Medical Research Library Building, Indianapolis, IN 46202 USA; 2 School of Optometry, Indiana University, 800 East Atwater Avenue, Bloomington, IN 47405 USA

**Keywords:** Cell death, Necroptosis

## Abstract

Chronic wounds show necroptosis from which keratinocytes must be protected to enable appropriate wound re-epithelialization and closure. Poloxamers, a class of synthetic triblock copolymers, are known to be effective against plasma membrane damage (PMD). The purpose of this study is to evaluate the efficacy of a specific poloxamer, surfactant polymer dressing (SPD), which is currently used clinically as wound care dressing, against PMD in keratinocytes. Triton X-100 (TX100) at sub-lytic concentrations caused PMD as demonstrated by the efflux of calcein and by the influx of propidium iodide and FM1-43. TX100, an inducer of necroptosis, led to mitochondrial fragmentation, depletion of nuclear HMGB1, and activation of signaling complex associated with necroptosis (i.e., activation of RIP3 and phosphorylation of MLKL). All responses following exposure of human keratinocytes to TX100 were attenuated by pre- or co-treatment with SPD (100 mg/ml). The activation and translocation of phospho-MLKL to the plasma membrane, taken together with depletion of nuclear HMGB1, characterized the observed cell death as necroptosis. Thus, our findings show that TX100-induced plasma membrane damage and death by necroptosis were both attenuated by SPD, allowing keratinocyte survival. The significance of such protective effects of SPD on keratinocytes in wound re-epithelialization and closure warrant further studies.

## Introduction

The chronic wound microenvironment is harsh often characterized by un-resolved inflammation, elevated protease activity, low pH, high peroxide levels and large masses of dead and dying cells^1–4^. Surgical debridement and negative pressure wound therapy are commonly used to cleanse the wound microenvironment^5–7^. After appropriate wound bed preparation^8^, wound dressings are intended to maintain favorable changes in the wound microenvironment such that the body’s healing responses may be effective in closing the wound^9–11^. In a wound defect on the skin, keratinocytes are the primary cells that achieve wound closure by re-epithelialization^12^. Thus, keratinocyte survival and migration are critically required to achieve this primary endpoint of wound healing. Wound dressings favoring keratinocyte survival in a hostile wound microenvironment are therefore of extraordinary significance.

At the open chronic wound-site, death of skin cells may be caused by a multitude of chemical mediators and mechanical injury. The chemical mediators in the form of pore-forming agents released by microbes directly compromise the integrity of the plasma membrane, potentially causing electrolyte imbalance and inflammasome activation^13–22^. On the other hand, chemical mediators in the form of reactive oxygen species, DAMPs (damage-associated molecular patterns), and cytokines emerging from the activity of pro-inflammatory cells breach plasma membrane integrity^17,23–29^. Such an insult is known to cause necroptosis (a programmed form of necrosis) in the chronic wound^27,30^. Previously, necrosis was considered as uncontrolled or accidental cell death caused by physical or chemical trauma. However, current evidence demonstrates necroptosis is regulated by intrinsic cellular proteins such as receptor interacting protein 3 (RIP3) and mixed lineage kinase domain-like (MLKL). Necroptosis is characterized by swelling and coalescing of intracellular organelles, loss of cellular ATP content, rupture of the plasma membrane, and subsequent leakage of cell contents^31,32^.

Non-ionic synthetic triblock copolymers, also known as poloxamers, are cognized for their function as membrane sealant^33,34^. Poloxamers have been evaluated against PMD in different pathological states, including respiratory dysfunctions, muscular dystrophy, sickle cell anemia, and neurodegenerative diseases such as Alzheimer's disease^33^. A particular triblock copolymer, Poloxamer 188, is the principal component of a wound dressing, viz., surfactant polymer dressing (SPD)^35^. Polymicrobial biofilm infection impairs functional wound closure as evident by deficient restoration of skin barrier function at the wound-site^36,37^. The anti-biofilm properties of SPD are, therefore, of value as wound care dressing^35^.

Complicated wounds are known to necrotic tissue burden which are clinically removed by surgical debridement and other such processes^36,38^. During such removal of dead tissue, it becomes important for a dressing like SPD to provide support to injured cells in a way that favors tissue repair. The site of injury is populated by a large number of skin cells that suffer from membrane damage secondary to infection and inflammation^39–43^. The effects of SPD on host skin cells remain unclear, however. In the experimental system studied, it has been our objective to test the effectiveness of SPD against PMD in keratinocytes to support membrane repair of injury affected cells such that they may participate in the overall process of skin repair. Triton X-100 (TX100) was recognized and utilized as a potent inducer of keratinocyte necroptosis at a sub-lytic dose employed.

## Results

### Triton X-100 induces cell death

**Figure 1:**
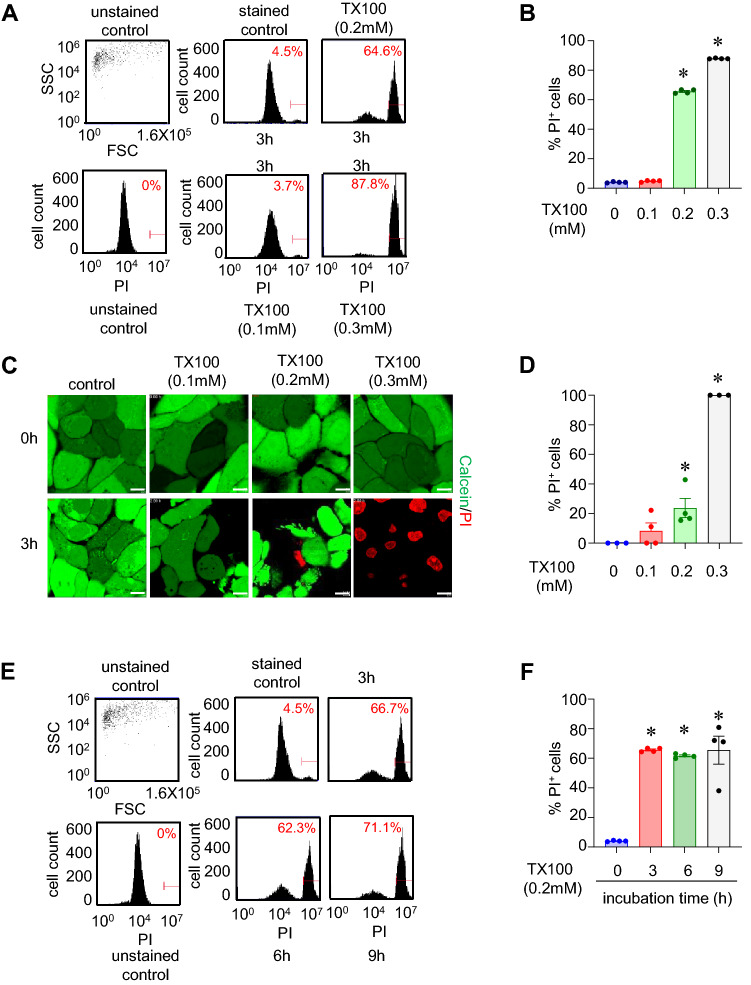
TX100-induced cell death is dose-dependent. Human keratinocytes (HaCaT) were exposed to TX100 at different concentrations and durations. Cells were stained with PI for flow cytometry and Calcein and PI for confocal microscopy. (**A**) Flow cytometry histograms showing %PI-positive cells upon treatment with increasing concentration (0.1 mM, 0.2 mM and 0.3 mM) of TX100 for 3 h. (**B**) Quantification of %PI-positive cells from flow cytometry representing mean ± SEM (n = 4). (**C**,**D**) Representative confocal images and quantification of %PI-positive cells after 3 h exposure to increasing levels of TX100. Scale, 10 µm. Data represent mean ± SEM (n = 3-4). (**E**,**F**) Flow cytometry measurements (%PI-positive cells) of HaCaT keratinocytes exposed to TX100 (0.2 mM) for 3, 6, and 9 h. Data represent mean ± SEM (n = 4). *p < 0.05 compared to control (TX100-untreated).

Cell death was assessed in human keratinocytes (HaCaT) by following the influx of PI and efflux of calcein as measured by flow cytometry and confocal microscopy. An increase in uptake of PI and efflux of calcein are markers of cell death. Exposure to 0.1, 0.2, and 0.3 mM of TX100 for 3 h led to  ~4%, ~60%, and ~85%, respectively (Fig. 1A,B) as measured by flow cytometry. The confocal images (Fig. 1C,D) supports the flow cytometry data. For further experiments, 0.2 mM of TX100 was chosen since this dose was sublytic and below the critical micellar concentration (CMC) of TX100. TX100-induced cell death was evident at 3 h, as observed by flow cytometry (Fig. 1E,F). Time-dependent exposure (3, 6, and 9 h) of TX100 did not increase the cell death (Fig. 1E,F).

### Triton X-100 induced cell death was blocked by pre- and co-treatment with SPD

**Figure 2:**
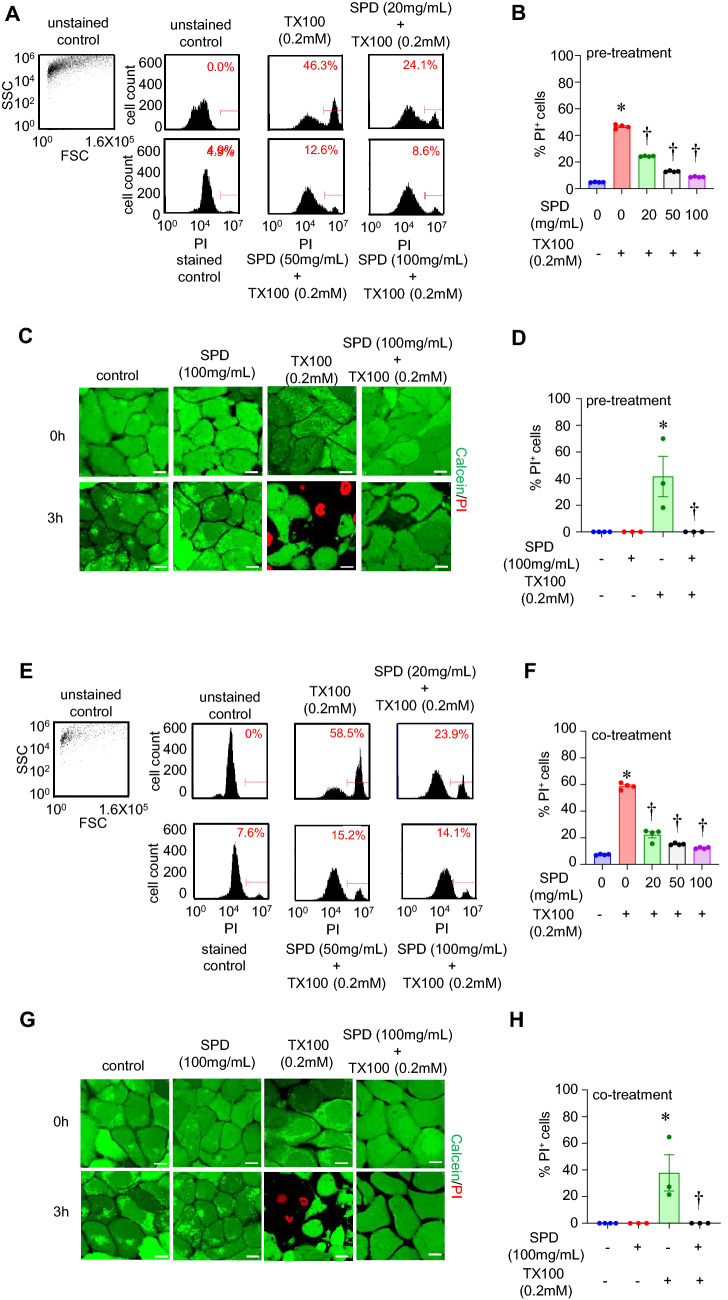
Pre- and co-treatment of human keratinocytes with SPD protected against TX100-induced cell death. (**A**–**D**) Human keratinocytes (HaCaT) were pretreated with SPD (100 mg/ml) for 24 h and then exposed to TX100 (0.2 mM) for 3 h in the continued presence of SPD (100 mg/ml). (**A**,**B**) Cells were stained with PI for flow cytometry. Flow cytometry histograms and quantification of %PI-positive cells. Data represent mean ± SEM (n = 4). *p < 0.05 compared to control (TX100-untreated). ^†^*p* < 0.05 compared to TX100-treated group. (**C**,**D**) Cells were stained with Calcein and PI for confocal microscopy. Representative images and quantification of %PI-positive cells are shown. Data represent mean ± SEM (n = 3-4). *p < 0.05 compared to control (TX100-untreated). ^†^*p* < 0.05 compared to TX100-treated group. (**E**–**H**) Human keratinocytes (HaCaT) were co-treated with SPD (100 mg/ml) and TX100 (0.2 mM) for 3 h. (**E**,**F**) Cells were stained with PI for flow cytometry. Flow cytometry histograms and quantification of %PI-positive cells. Data represent mean ± SEM (n = 4). *p < 0.05 compared to control (TX100-untreated). ^†^*p* < 0.05 compared to TX100-treated group. (**G**, **H**) Cells were stained with Calcein and PI for confocal microscopy**.** Representative images and quantification of %PI-positive cells are shown. Scale, 10 µm. Data represent mean ± SEM (n = 3-4). *p < 0.05 compared to control (TX100-untreated). ^†^*p* < 0.05 compared to TX100-treated group.

**Figure 3:**
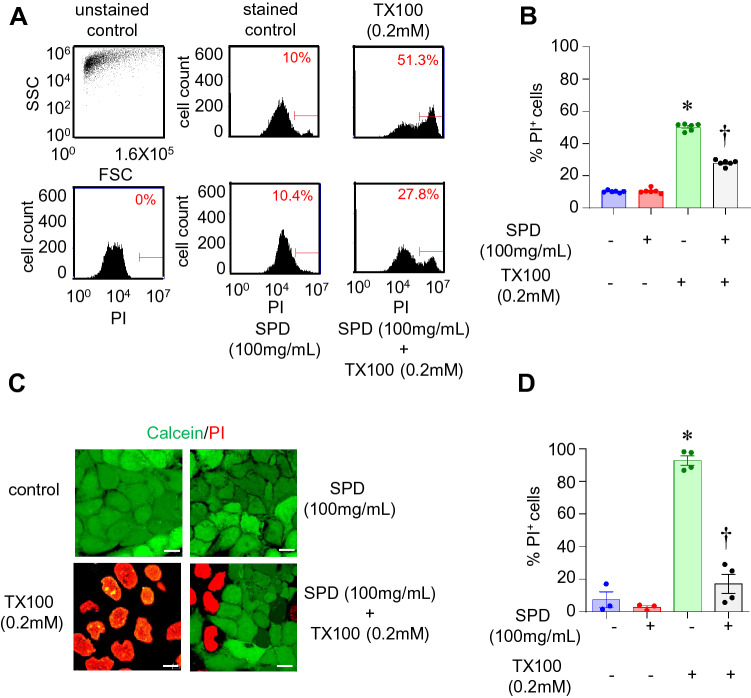
Prolonged efficacy of SPD against TX100-induced cell death. Human keratinocytes (HaCaT) were first co-treated with SPD (100 mg/ml) and TX100 (0.2 mM) for 3 h. Cells were subsequently washed with PBS and incubated in a fresh medium for 24 h containing SPD and then analyzed for cell death by flow cytometry and confocal microscopy. (**A**,**B**) Flow cytometry histograms and quantification of %PI-positive cells. Data represent mean ± SEM (n = 6). *p < 0.05 compared to control (TX100-untreated). ^†^*p* < 0.05 compared to TX100-treated group. (**C**,**D**) Confocal microscopy images and quantification of %PI-positive cells. Scale, 10 µm. Data represent mean ± SEM (n = 3–4). *p < 0.05 compared to control (TX100-untreated). ^†^*p* < 0.05 compared to TX100-treated group.

Exposure to SPD alone was not toxic to cells at concentrations of up to 100 mg/ml (Fig. S1). To assess the efficacy of SPD against TX100-induced cell death, HaCaT cells were pre-treated with SPD at different concentrations (20, 50, and 100 mg/ml) for 24 h and then exposed cells to TX100 in the continued presence of SPD. Data from flow cytometry (Fig. 2A,B) and confocal microscopy (Fig. 2C,D) demonstrate that SPD consistently reduced cell death in response to 3 h exposure to TX100 at 0.2 mM. Similar findings were observed in human epidermal keratinocytes (HEKa) cells, where pre-treatment with SPD at a concentration of 100 mg/ml was able to reduce TX100-induced cell death (Fig. S2). In addition, HaCaT cells were co-treated with TX100 and SPD simultaneously (without pre-treatment with SPD), and cell death was analyzed by flow cytometry and confocal microscopy. Co-treatment with SPD was effective against TX100-induced cell death (Fig. 2E–H). To test if the protective efficacy of SPD over TX100-induced cell death persisted long-term, HaCaT cells were co-treated with TX100 and SPD (100 mg/ml) for 3 h, washed off, and then subjected to analysis of cell death after 24 h. Interestingly, TX100-induced cell death was significantly less when co-treated with SPD (Fig. 3A–D).

### SPD maintained the integrity of the plasma membrane following exposure to Triton X-100

**Figure 4:**
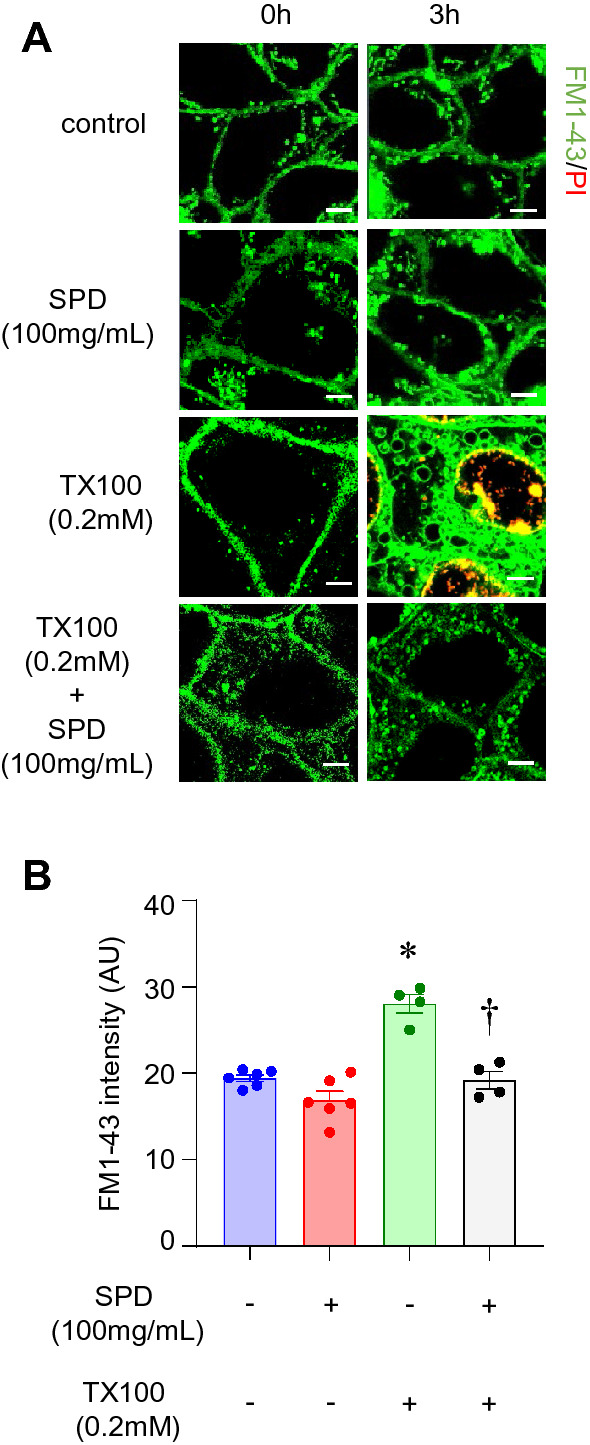
SPD protects against TX100-induced plasma membrane damage. Human keratinocytes (HaCaT) were exposed to TX100 (0.2 mM) for 3 h in presence of SPD (100 mg/ml). Cells were stained with FM1-43 and subjected to microscopy. (**A**,**B**) Representative microscopic images and quantification of intensity of FM1-43 was assessed by confocal microscopy. Scale, 5 µm. Data represent mean ± SEM (n = 4–6). *p < 0.05 compared to control (TX100-untreated). ^†^*p* < 0.05 compared to TX100-treated group.

Exposure to TX100 resulted in intense staining in the cytoplasm, indicating movement of the dye (FM1-43) and subsequent intercalation into membranes of the cytoplasmic organelles (Fig. 4A,B). The accumulation was significantly attenuated in the presence of SPD (Fig. 4A,B).

### SPD reduced nuclear depletion of HMGB1 and mitochondrial fragmentation

**Figure 5:**
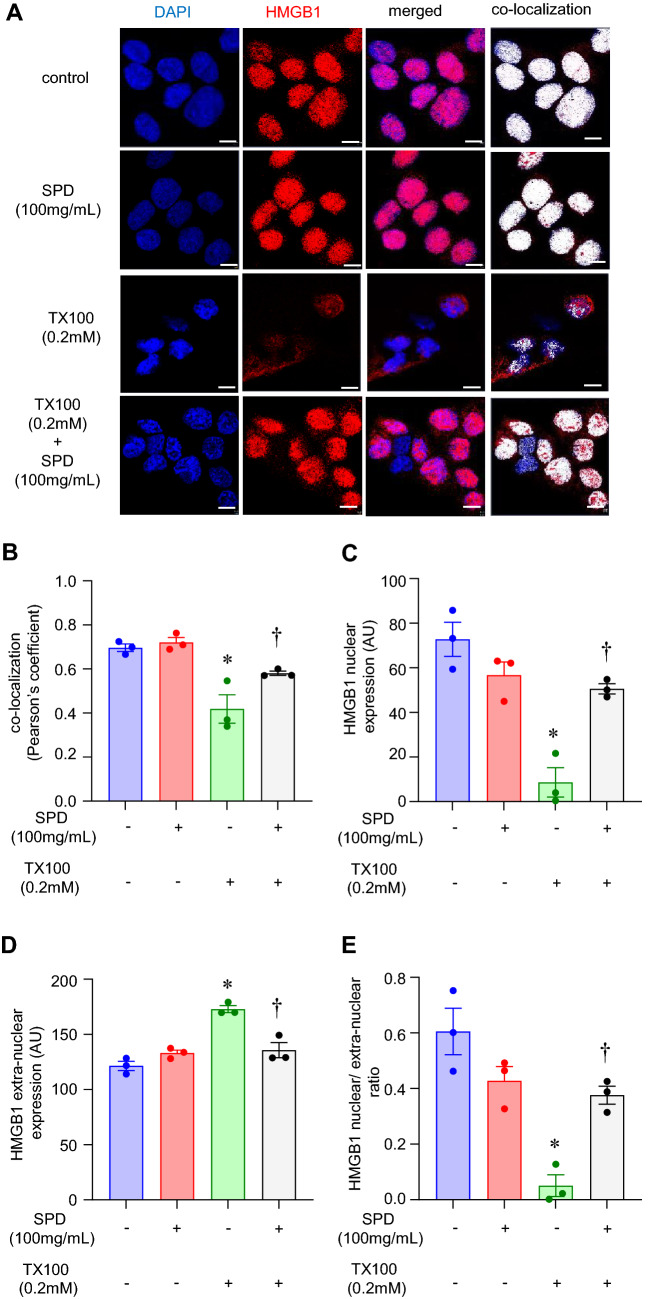
SPD rescued TX100-induced nuclear depletion of HMGB1. Human keratinocytes (HaCaT) were exposed to TX100 (0.2 mM) for 3 h in presence of SPD (100 mg/ml). Cells were then fixed and subjected to immunocytochemistry using HMGB1 antibody and DAPI as nuclear stain (**A**) Representative microscopic images of HMGB1 (red) and DAPI (blue) as assessed by immunocytochemistry in combination with confocal microscopy. Co-localization was performed using Zen Blue software (v.3.1). Scale, 10 µm. (B) Co-localization of DAPI and HMGB1 as assessed by Pearson’s coefficient. Data represent mean ± SEM. (n = 3). *p < 0.05 compared to control (TX100-untreated). ^†^*p* < 0.05 compared to TX100-treated group. (**C**,**D**) Quantification of nuclear and extranuclear HMGB1 was analyzed by Zen Blue software (v.3.1). Data represent mean ± SEM. (n = 3). *p < 0.05 compared to control (TX100-untreated). ^†^*p* < 0.05 compared to TX100-treated group. (**E**) Ratio of nuclear to extranuclear HMGB1. Data represent mean ± SEM. (n = 3). *p < 0.05 compared to control (TX100-untreated). ^†^*p* < 0.05 compared to TX100-treated group.

**Figure 6:**
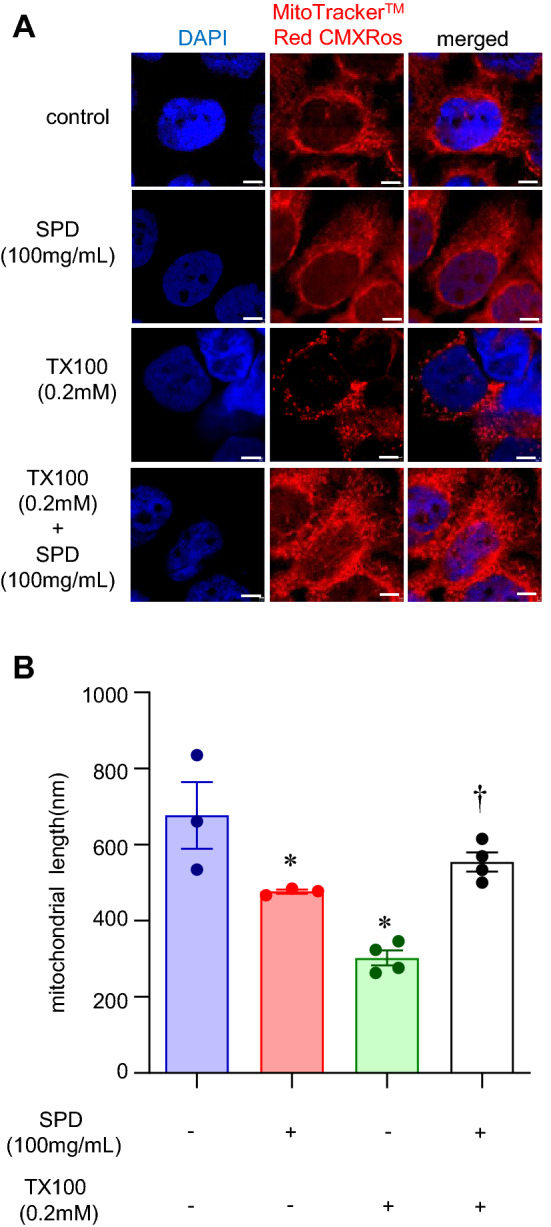
SPD rescued TX100-induced mitochondrial fragmentation. Human keratinocytes (HaCaT) were exposed to TX100 (0.2 mM) for 1 h in presence of SPD (100 mg/ml). Cells were then stained with MitoTrackerRedCMXRos. (**A**,**B**) Representative microscopic images and quantification of tubular length of the mitochondria was assessed by confocal microscopy for at least 100 mitochondria selected from the three different regions of interests per sample. Scale, 10 µm. Data represent mean ± SEM (n = 3–4). *p < 0.05 compared to control (TX100-untreated). ^†^*p* < 0.05 compared to TX100-treated group.

High mobility group protein B1 (HMGB1) is a nuclear protein vital for the maintenance of several nuclear functions, including replication, recombination, transcription, and DNA repair^44,45^. Nuclear depletion of HMGB1 is a marker of necroptosis^46^. We sought to assess the nuclear abundance of HMGB1 post-insult. Immunocytochemistry with an antibody specific to HMGB1 was performed to assess its cellular localization. Nuclear depletion of HMGB1, a classical marker of necroptosis, was observed in response to TX100 (Fig. 5A–E, S3). In agreement with protection against cell death, SPD at 100 mg/ml prevented the nuclear depletion of HMGB1 (Fig. 5A–E, S3). Yet another important role ascribed to HMGB1 is in mitochondrial quality control^47^. Thus, we examined if the mitochondria are influenced concomitant with HMGB1 depletion. For this purpose, cells were treated with SPD and TX-100 for 1 h, followed by staining with MitoTrackerRedCMXRos. Exposure to TX100 at 0.2 mM led to a fragmentation of the tubular mitochondria (Fig. 6A,B). The reduced length of mitochondria in response to TX100 was blocked by co-treatment with SPD (Fig. 6A,B).

### Triton X-100 induced upregulation of RIP3 kinase as well as activation of MLKL

**Figure 7:**
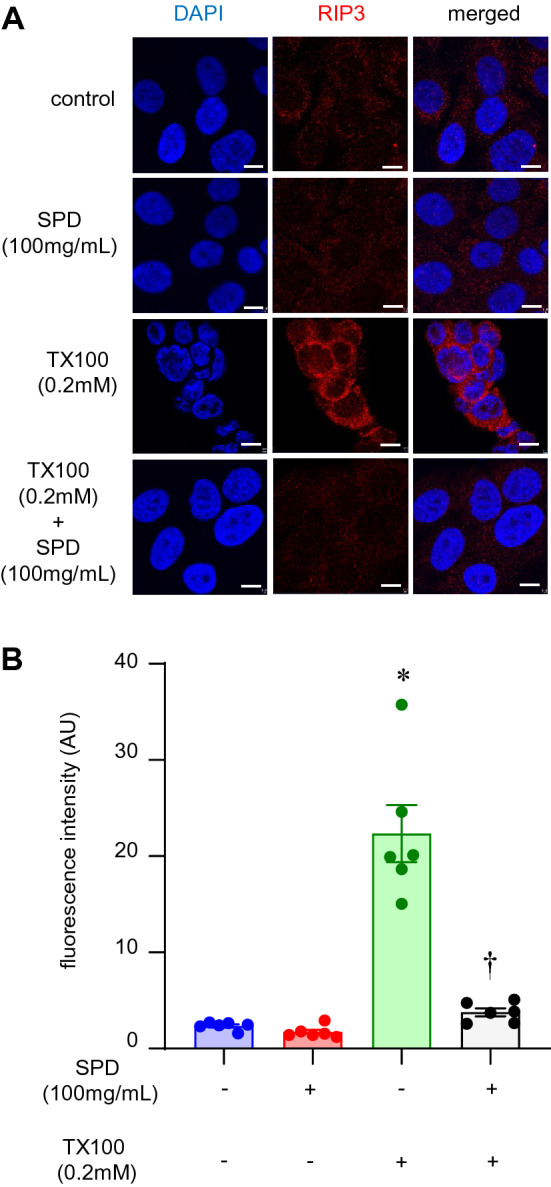
SPD blunted TX100-induced RIP3 expression. Human keratinocytes (HaCaT) were exposed to TX100 (0.2 mM) for 3 h in presence of SPD (100 mg/ml). Cells were then fixed and immunostained with RIP3 antibody. (**A**,**B**) Representative microscopic images and quantification of RIP3 expression was assessed by confocal microscopy. Scale, 10 µm. Data represent mean ± SEM (n = 6). *p < 0.05 compared to control (TX100-untreated). ^†^*p* < 0.05 compared to TX100-treated group.

**Figure 8:**
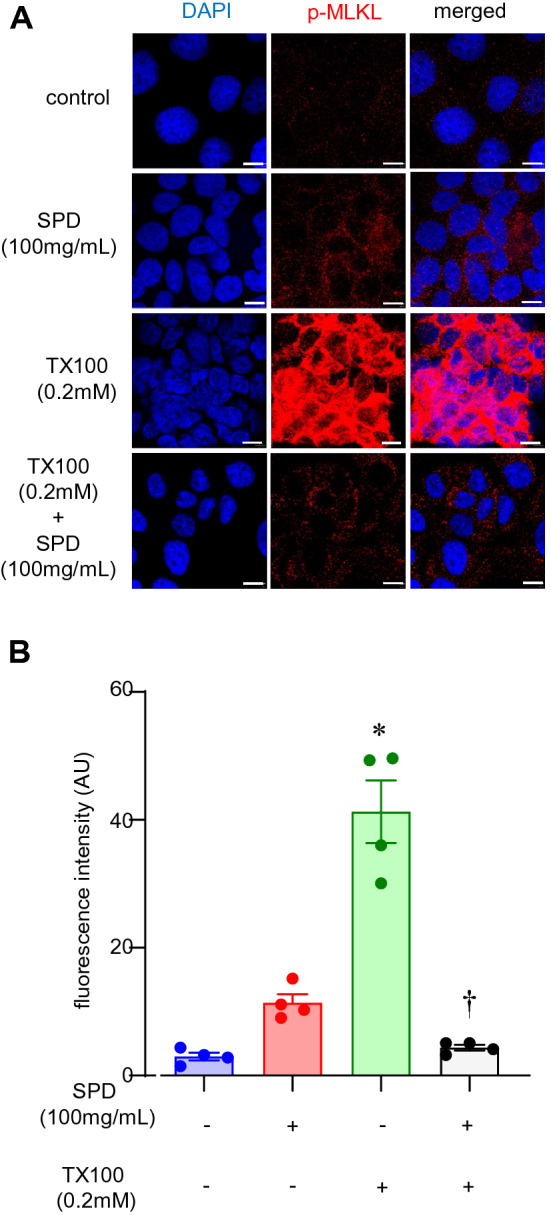
SPD blunted TX100-induced phosphorylation of MLKL. Human keratinocytes (HaCaT) were exposed to TX100 (0.2 mM) for 3 h in presence of SPD (100 mg/ml). Cells were then fixed and immunostained with phospho-MLKL antibody. (**A**,**B**) Representative microscopic images and quantification of phospho-MLKL was assessed by confocal microscopy. Scale, 10 µm. Data represent mean ± SEM (n = 4). *p < 0.05 compared to control (TX100-untreated). ^†^*p* < 0.05 compared to TX100-treated group.

Among the patterns of cell death in response to TX100, an involvement of apoptosis, including the canonical pathways, has been questioned^48–50^. Since there is a potential for the release of DAMP (such as HMGB1), we examined programmed necrosis, i.e., necroptosis, following treatment with TX100. Here, we focused on the signaling complex upstream of necroptosis. As a first step, we tested RIP3 expression by immunocytochemistry after TX100 treatment. Exposure to TX100 for 3 h led to an increase of RIP3, which was attenuated by the co-treatment with SPD (Fig. 7A,B). To test the role of MLKL, we performed immunocytochemistry of phospho-MLKL. MLKL was found to be phosphorylated upon treatment with TX100, which was significantly attenuated by co-treatment with SPD (Fig. 8A,B).

## Discussion

SPD is employed as a water-soluble burn wound dressing and is FDA cleared^51,52^. SPD is not only well tolerated by patients, but anecdotal clinical evidence show beneficial effects in wound healing^53,54^. It is therefore of extraordinary significance to understand the mechanism of action of SPD. Having a hydrophilic polymer chain, SPD can form a reservoir for topical hydrophilic antimicrobials agents^55^. SPD can be removed from wound beds easily compared to standard silver sulfadiazine creams on the burn wounds. They can effectively disrupt bacterial biofilm infection^35^. Despite several advantages, cellular mechanisms contributing to the efficacy of SPDs on cytoprotection under conditions inciting PMD remain yet to be fully resolved. During PMD, necroptosis form of cell death is evident at the site of chronic wounds^56^. The present work characterizes TX100 as a potent inducer of necroptosis in human keratinocytes. At dosages achievable at the wound site, where SPD is delivered topically, SPD prevented necroptosis induced by TX100. Any sparing of keratinocyte death at the hostile wound microenvironment is likely to facilitate wound-re-epithelialization and therefore closure.

As strategies to induce reproducible PMD, many approaches have been reported^57–62^. They include (a) laser-induced membrane damage, (b) exposure to pore-forming pathogenic cytotoxins, (c) mechanical injury by glass beads, shear stress, cellular contraction, cell scraping, and forcing cells through a needle, and (d) exposure to detergents^63,64^. Of these methods, the pore-forming toxins and detergents are suitable for induction of PMD simultaneously in a large number of cells^63^, and hence are highly suitable for high-throughput screening of potential therapeutic agents. However, in the case of bacterial cytotoxins, a significant disadvantage is several toxins would be required to produce membrane pores of varying sizes. With detergents, on the other hand, the extent of PMD can be tuned by varying their concentration and time of exposure. These variables can easily be controlled during the conduct of experiments. Controlled exposure to TX100, a non-ionic detergent, is widely employed in cell biological applications^50,65,66^. The interactions of TX100 with the plasma membrane in other cell types have been partly characterized. In particular, at sub-lytic doses, TX100 is known to permeabilize the plasma membrane and cause cell death by mechanisms not yet established^48–50,65–67^. The sub-lytic dose, however, corresponds to levels below CMC, which is ~ 0.24 mM^48^. Because of their polar head group, TX100 molecules disrupt hydrogen bonding present within the cell’s lipid bilayer and lead to the destruction of the compactness and integrity of lipid membrane^65^. Cells undergo irreversible permeabilization of the membrane and structural collapse when the TX100 concentration reached the CMC^65^. The action of TX100 also depends on the composition of the lipid bilayer^68^. TX100 action starts by destabilization of the lipid component of the membranes (non-cooperative binding of detergents to the membrane to the cooperative binding). This leads to the formation of membrane fragments of proteins and lipids with detergent shielded edges^69^. The changes in plasma membrane permeability and fluidity also plays a critical role in psoriasis^70^.

The non-ionic detergent, TX100, is known to elicit plasma membrane damage (demonstrated usually by transiently increased membrane permeability) and subsequent cell death^48–50,66,67,71,72^. In some cells, TX100 induces apoptotic cell death^48–50,66,67^. Lipid homeostasis of the skin is unique such that lipid signaling is a key contributor to the turn-over of skin cells and the lipid composition of the skin contributes to its barrier function^73^. It is therefore not surprising that keratinocytes respond differently to detergents. For example, NP-40 is a non-ionic detergent like TX100 yet it induces necrosis in HaCaT keratinocytes^74^. Our choice to make use of TX100 to induce experimental PMD turned out to be a useful strategy to produce damage in a large number of cells simultaneously. At sub-lytic concentrations, TX100 produced transient membrane permeabilization without causing immediate cell death. Such membrane permeabilization is known to be a characteristic feature of necroptosis^29^. SPD, by itself, showed no cytotoxicity. When co-treated to cells and subsequently removed, SPD retained its cytoprotective properties indicating of successful cellular uptake by a magnitude that was functional relevant. Cytoprotective effects of SPD co-treated with TX100 demonstrated that SPD is not only safe on its own but that it did not chemically interact with TX100 in a harmful manner.

FM1-43 is a fluorescent membrane probe^75^. It is not fluorescent in an aqueous medium. However, the dye becomes intensely fluorescent when it intercalates into the outer leaflet of the plasma membrane^75^. The fluorescence of FM1-43 is only at the membrane. An increase in fluorescence is an indicator of plasma membrane damage^76^. In keratinocytes, TX100 dependent increase in FM1-43 fluorescence establishes the ability of the detergent to cause PMD. PMD is known to occur during necroptosis^29^. This work is the first to recognize the value of FM1-43 as a productive approach to detect early necroptosis events. Understanding of mitochondrial changes during the course of necroptosis has led to the observation that the RIP1-RIP3 complex initiates mitochondrial fission^77^. Co-ordinated with DNA replication, mitochondrial fission divides the organelle. In a healthy cell, mitochondria exist as a dynamic network of fission and fusion^78^. During necroptosis, the balance favors fission and manifests as reduced mitochondrial length^79^. MitoTrackerRedCMXRos is a cationic dye, which accumulates in the mitochondrial membrane of living cells^80^. It is intrinsically fluorescent, binds irreversibly to the polarized mitochondrial membrane, and does not require reduction or oxidation for the emission of fluorescence^80^. Thus, it is well suited to study mitochondrial fission during necroptosis. TX100 dependent reduction in mitochondrial length, indicative of fission, was completely spared in the presence of SPD.

HMGB1 is a ubiquitous nuclear protein, the release of which is a characteristic feature of necroptosis^46^. Nuclear HMGB1 is not only released as a DAMP but is also associated with mitochondrial quality control^47^. Loss of nuclear HMGB1 is known to cause mitochondrial fission^81,82^. In the nucleus, the cell-survival functions of HMGB1 are attributed to its ability to facilitate assembly of DNA binding proteins on chromatin^83^. Once released from the nucleus, HMGB1 interacts with specific TLRs and incites local inflammation^84–86^. Thus, the release of nuclear HMGB1 is counterproductive to the wound healing cascade where timely resolution of inflammation is critical^2^. TX100 caused rapid and overt depletion of nuclear HMGB1. When expressed as the ratio of nuclear:extranuclear HMGB1, TX100 caused marked depletion which was significantly rescued under conditions of SPD co-treatment.

The canonical pathway for the induction of necroptosis entails activation of RIP1K-RIP3K, followed by activation of phospho-MLKL. Activation of RIP1K, a serine-threonine kinase causes oligomerization and autophosphorylation of RIP3K at Ser227^87–90^. RIP3K, thus activated, drives phosphorylation of MLKL^28,87,91,92^. The phosphorylated MLKL is translocated to the plasma membrane leading to pore formation reminiscent of gasdermins in pyroptosis^28,87,91,92^. As a pseudokinase, MLKL does not target any protein for phosphorylation. Instead, upon activation, MLKL is trafficked to the plasma membrane^28,31,87,93^. Upon interaction with inositide lipids associated with the plasma membrane, MLKL induces necroptosis causing cell death^91^. In particular, MLKL at the plasma membrane form pores that are directly implicated in cell death. In this work, TX100 activated RIP3 and generated phospho-MLKL. Moreover, in agreement with the spared depletion of nuclear HMGB1, the abundance of both RIP3 and phospho-MLKL were blunted in the presence of SPD. Inhibition of keratinocyte necroptosis is known to be a protective effect against psoriatic inflammation^94^. The clinical relevance of SPD may thus be broader than the realm of chronic wound management.

Taken together, this work shows that SPD, clinically used as wound care dressing, is likely to have functions beyond its reported effects on biofilm management. Necroptotic death, known to be abundant in chronic wounds, can be managed by SPD. The effects of this poloxamer are evident in multiple signaling events leading to necroptosis. Sparing of keratinocyte nuclear HMGB1 is likely not only to spare these cells enabling re-epithelialization, but also to manage chronic inflammation caused by release of this DAMP molecule. Additional studies testing these multifaceted effects of SPD in a chronic wound setting are thus warranted.

## Materials and methods

### Surfactant polymer dressing

PluroGel (Medline Industries, Inc. Northfield, IL), which contains Poloxamer 188, was employed as SPD in all the experiments. SPD was reconstituted to 1 g/ml in Dulbecco's phosphate-buffered saline (DPBS, Gibco, Carlsbad, CA # 14190250) to make the stock solution which was then filtered using 0.2 µm filter. Further, it was diluted in media and used to treat the cells.

### Cell culture

Immortalized human keratinocytes (HaCaT) cells were grown at 37 °C temperature in a humidified atmosphere consisting of 95% O_2_ and 5% CO_2_ in Dulbecco's modified Eagle's medium (DMEM, low glucose, Gibco, Carlsbad, CA # 11885092) that was supplemented with 10% FBS (Gibco, Carlsbad, CA), 100 IU/ml penicillin, and 0.1 mg/ml streptomycin^37,95,96^.

Primary Human Epidermal Keratinocytes (HEKa) cells (ATCC, Manassas, VA #PCS-200-011) were cultured in dermal cell basal media (ATCC, Manassas, VA #PCS-200-030) supplemented with human keratinocyte growth supplement (ATCC, Manassas, VA #PCS-200-040) and Penicillin–Streptomycin–Amphotericin B solution (ATCC, Manassas, VA #PCS-999-002) and incubated at 37 °C in a CO_2_ incubator. After every 48 h, the media was changed until the cells reached 80–90% confluency. HEKa cells would start to become senescent at about passage 3 or 4. Therefore, all the experiments were performed with cells at passages 2 to prevent cell senescence.

### Characterization of cell death by flow cytometry

Cells were grown in twelve-well plates at a seeding density of 0.1million/ml. After reaching confluence, typically after 24 h, cells were treated with TX100 with and without SPD. Cell viability was measured using propidium iodide staining as previously described^97–99^. After the treatments, floating dead cells were collected from the medium. The attached cells were trypsinized using trypsin–EDTA (Gibco, Carlsbad, CA #15400054) and then collected into the 4% FBS. The suspension of dead cells was centrifuged at 15,000*g* for 10 min, while the suspension of trypsinized cells was centrifuged at ~ 700*g* for 10 min. Subsequently, the pellets were mixed in a 200 µL of PI (10 µg/ml, diluted in 4% FBS) and incubated at room temperature for 15 min. The flow cytometry measurements were performed using the BD Accuri flow cytometry (BD Biosciences, USA) and analyzed as previously described^100,101^.

### Characterization of cell death by confocal microscopy

Cells were grown in the 35 mm Quad µ-Dish (ibidi, Gräfelfing, Germany # 80416). After reaching confluence, typically after 24 h, cells were treated with TX100 with and without SPD containing calcein-AM (Life Technologies, Carlsbad, CA # C3099) and propidium iodide (Sigma-Aldrich, St. Louis, MO # P4170). The confocal imaging was performed at 0 h and 3 h.

### Immunocytochemistry

Cells were grown and treated in the 8-well chamber slides (Thermo Scientific Nunc Lab-Tek, Waltham, MA, # 12-565-22). The attached cells were washed with DPBS and were subjected to immunocytochemistry as previously described^3,37,100,101,102^. Subsequently, cells were fixed with intracellular (IC) fixation buffer (eBioscience, San Diego, CA, # 00-8222-49) followed by the permeabilization using 0.1% Triton X-100^3,37,100,101,102^. The cells were incubated with 10% normal goat serum (NGS, Vector Labs, Inc, Burlingame, CA, # S-1000) for 1 h at room temperature for blocking non-specific binding of antibodies^3,37,100,101,102^. Next, cells were exposed to the primary antibodies made in 2.5% NGS overnight at 4 °C. Following antibodies have been used: HMGB1 antibody (Cell Signaling Technology, Inc., Danvers, MA, # 3935S), RIP3 antibody (Cell Signaling Technology, Inc., Danvers, MA, # 13526S), MLKL (phospho-Ser358) antibody (Biorbyt, Cambridge, UK # orb499999). The cells were washed with PBST for 5 min (twice) followed by the incubation with a secondary antibody made in 2.5% NGS for 1 h at room temperature in the dark^3,37,100,101,102^. The cells were again washed with PBST, followed by the staining with DAPI (Thermo Scientific, Waltham, MA # 62248) and mounted with mounting media (Vector Labs, Inc, Burlingame, CA # H-1400)^3,37,100,101,102^. The microscopic images were captured with a Zeiss LSM 880 Airyscan super-resolution confocal microscope^100^. For mitochondrial staining, the cells were incubated with MitoTrackerRedCMXRos (Invitrogen, Carlsbad, CA # M7512) for 30 min before fixation. Intensity and Pearson’s coefficient were analyzed using ImageJ and Zen Blue software (v.3.1), respectively^96,100^. The nuclear and extranuclear localization of HMGB1 was quantified using Zen Blue software (v.3.1). Briefly, a colocalized image of HMGB1 with DAPI was extracted by masking the extranuclear HMGB1 (no colocalized with DAPI. Extranuclear HMGB1 was quantified by the following formula:

$${\text{Extranuclear}}\,{\text{HMGB}}1\,{\text{intensity}} = {\text{Total}}\,{\text{HMGB}}1\,{\text{intensity}}{-}{\text{masked}}\,{\text{HMGB}}1\,{\text{intensity}}.$$

### Statistical analyses

All the data are reported as mean ± SEM of three to nine experiments, as indicated in respective figure legends. One-way ANOVA was used to determine significant differences. A *p* value < 0.05 was considered statistically significant.

## Supplementary Information

Supplementary Figures.

## Data Availability

All data generated or analyzed during this study are included in this published article (and its Supplementary Information files).
