# Supplementary material for: A surfactant polymer wound dressing protects human keratinocytes from inducible necroptosis
Source: Sci Rep. 2021 Feb 23;11:4357. doi: 10.1038/s41598-021-82260-x (PMC7902632; doi:10.1038/s41598-021-82260-x)
Supplement: Supplementary file 1 — Supplementary Figures. [file 41598_2021_82260_MOESM1_ESM.pdf]

A SURFACTANT POLYMER WOUND DRESSING PROTECTS HUMAN KERATINOCYTES FROM INDUCIBLE NECROPTOSIS

Puneet Khandelwal[1*], Amitava Das[1*], Chandan K. Sen[1], Sangly P. Srinivas[2], Sashwati Roy[1] and Savita Khanna[1#]

[1]Department of Surgery, Indiana Center for Regenerative Medicine and Engineering, Indiana University School of Medicine, Indianapolis, IN, 46202. [2]School of Optometry, Indiana University, 800 East Atwater Avenue, Bloomington, Indiana, 47405, USA. [#]Corresponding author.

* *these authors contributed equally*

Running Title: Surfactant Polymer Dressing against cell death

Address correspondence to:

Savita Khanna, PhD

975 W Walnut St,

Medical Research Library Building,

Indiana University School of Medicine

Indianapolis, IN 46202.

Tel. 317 278 2736

E-mail: sjkhanna@iu.edu

Supplementary Figure Legends

Figure S1. SPD is not cytotoxic to human keratinocytes (HaCaT).

HaCaT cells were treated with SPD (0, 20, 50, 100, 200, and 400mg/mL) for 24h. Cells were stained with PI for flow cytometry. Flow cytometry histograms and quantification showing %PI-positive cells upon treatment with increasing concentration (0, 20, 50, 100, 200, and 400mg/mL) of SPD for **(A,B)** 6h, **(C,D)** 12h, and **(E,F)** 24h. Data represent mean ± SEM. (n=4-8). *$p<0.05$ compared to untreated control.

Figure S2. Pre-treatment of primary human epidermal keratinocytes (HEKa) with SPD protected against TX100-induced cell death.

HEKa cells were pretreated with SPD (100mg/mL) for 24h and then exposed to TX100 (0.2mM) for 3h in the continued presence of SPD (100mg/mL). Cells were stained with PI for flow cytometry. Flow cytometry histograms and quantification of %PI-positive cells. Data represent mean ±SEM (n=8-9). *$p<0.05$ compared to control (TX100-untreated). †$p<0.05$ compared to TX100-treated group.

Figure S3. Representative images of quantification of co-localization.

Co-localization was measured using Zen Blue software (v.3.1).

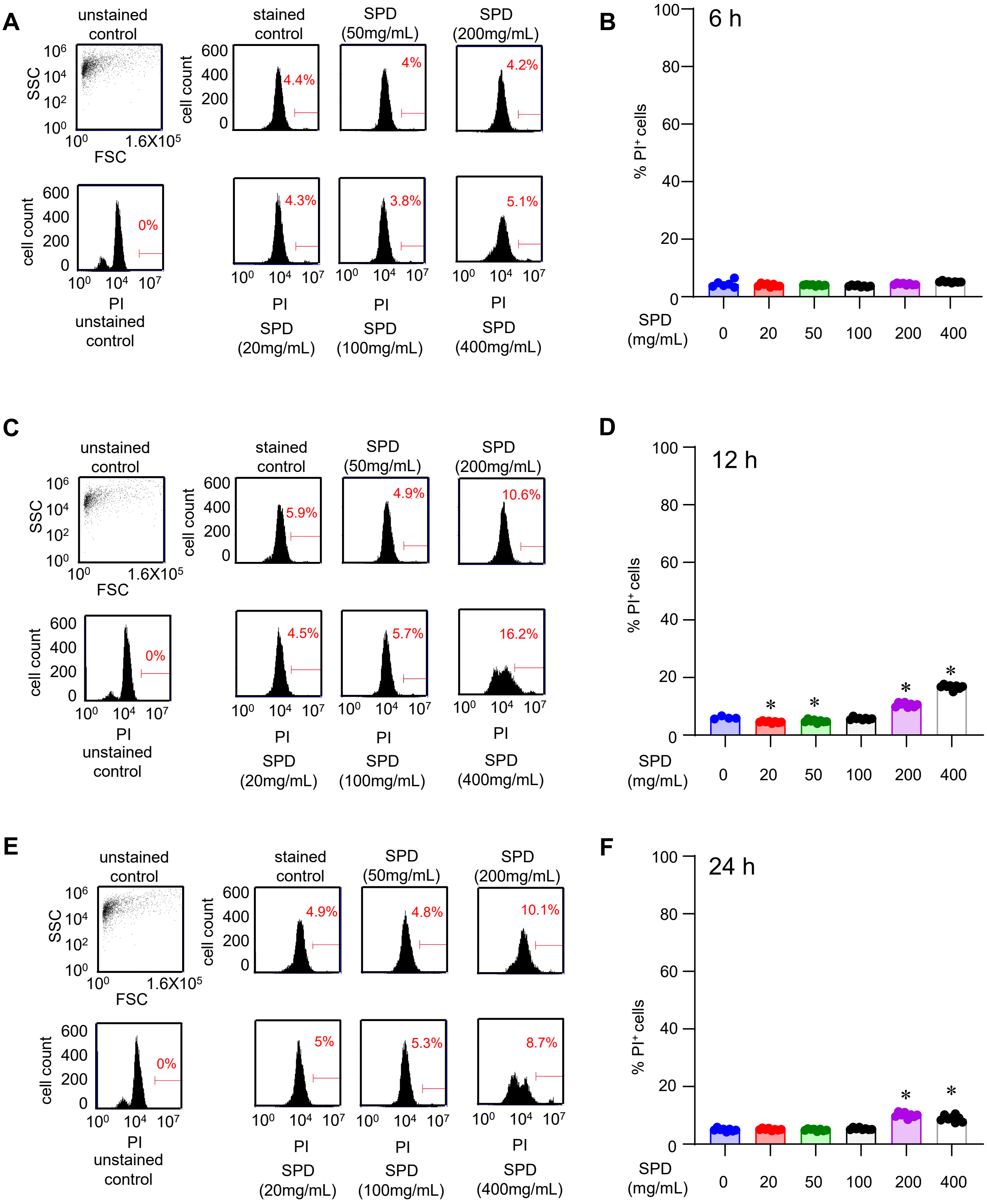
A
unstained control
SSC
FSC
stained control
SPD (50mg/mL)
SPD (200mg/mL)
cell count
4.4%
4%
4.2%
0%
4.3%
3.8%
5.1%
PI
unstained control
SPD (20mg/mL)
SPD (100mg/mL)
SPD (400mg/mL)
B
6 h
% PI+ cells
SPD (mg/mL)
0
20
50
100
200
400
C
5.9%
4.9%
10.6%
4.5%
5.7%
16.2%
D
12 h
E
4.9%
4.8%
10.1%
5%
5.3%
8.7%
F
24 h

Fig S1

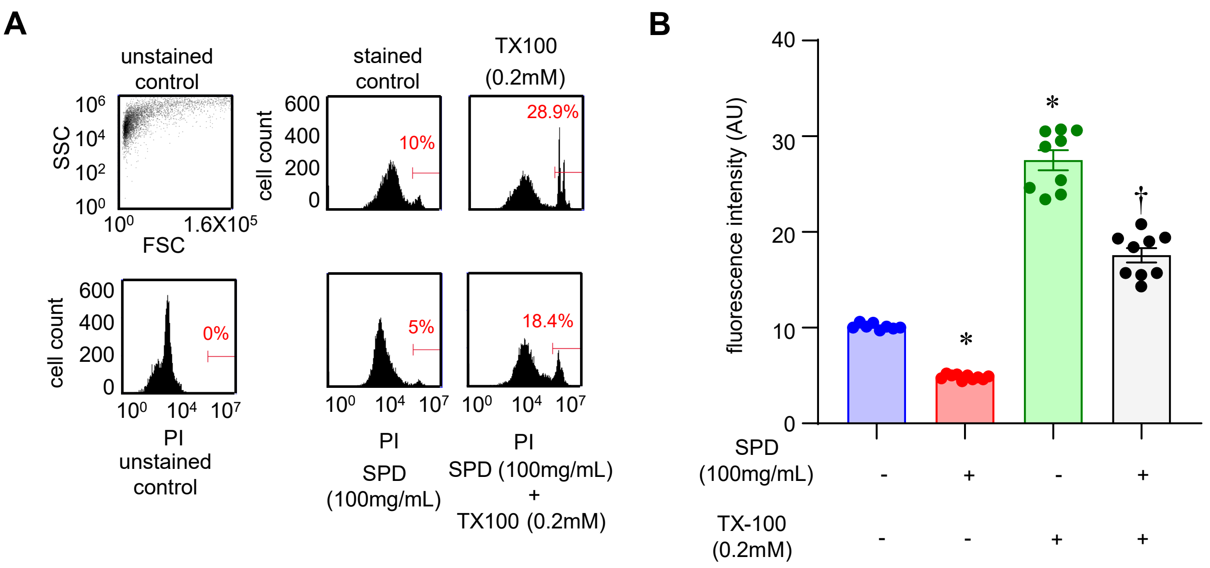
A
unstained control
SSC
FSC
10^6
10^4
10^2
10^0
1.6X10^5
stained control
TX100 (0.2mM)
cell count
600
400
200
0
10%
28.9%
0%
5%
18.4%
10^7
PI
unstained control
SPD (100mg/mL)
SPD (100mg/mL) + TX100 (0.2mM)
B
fluorescence intensity (AU)
40
30
20
10
*
†
SPD (100mg/mL)
TX-100 (0.2mM)
-
+

Fig S2

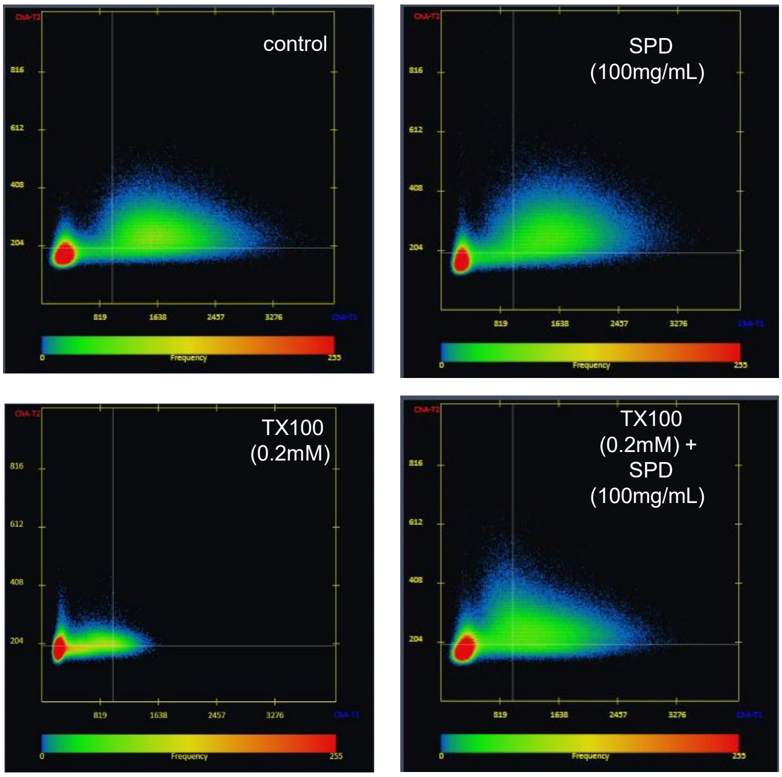

Fig S3
